# Human immunodeficiency virus, hepatitis C, and inflammatory biomarkers in individuals with alcohol problems: a cross-sectional study

**DOI:** 10.1186/1471-2334-13-399

**Published:** 2013-08-29

**Authors:** Kaku A Armah, Emily K Quinn, Debbie M Cheng, Russell P Tracy, Jason V Baker, Jeffrey H Samet, Matthew S Freiberg

**Affiliations:** 1 Department of Epidemiology, University of Pittsburgh Graduate School of Public Health, Pittsburgh, PA, USA; 2 Data Coordinating Center, Boston University School of Public Health, Boston, MA, USA; 3 Clinical Addiction Research and Education (CARE) Unit, Section of General Internal Medicine, Boston Medical Center and Boston University School of Medicine, Boston, MA, USA; 4 Department of Biostatistics, Boston University School of Public Health, Boston, MA, USA; 5 Departments of Pathology and Biochemistry, College of Medicine, University of Vermont, Burlington, VT, USA; 6 Department of Medicine, University of Minnesota, Hennepin County Medical Center, Minnesota, MN, USA; 7 Department of Community Health Sciences, Boston University School of Public Health, Boston, MA, USA; 8 Division of General Internal Medicine, Center for Research on Health Care, University of Pittsburgh Medical Center, 230 McKee Pl, Suite 600, Pittsburgh, PA 15213, USA

**Keywords:** HIV, HCV, Inflammation, Alcohol, Liver, Comorbidity

## Abstract

**Background:**

Assessing whether hepatitis C (HCV) co-infection with human immunodeficiency virus (HIV) is associated with increased inflammation is complex. The liver, integral to inflammatory biomarker synthesis, is compromised by HCV and alcohol abuse. Using single liver-synthesized biomarkers (e.g. C-reactive protein) to represent inflammation may not be appropriate in HIV/HCV co-infection. We hypothesized that 1) detectable HIV/HCV RNA was independently associated with increased inflammation; 2) a composite inflammation measure describes inflammation differently from single inflammatory biomarkers.

**Methods:**

We compared inflammation by HIV/HCV group in a cohort of 361 HIV infected participants from the HIV-Longitudinal Interrelationships of Viruses and Ethanol study. Inflammatory biomarkers >75th percentile were considered elevated. Associations between HIV/HCV group and elevated biomarkers were analyzed as a composite measure (inflammatory burden) or individually. We defined inflammatory burden as number of concurrently elevated biomarkers. Biomarkers included interleukin-6 (IL-6), C-reactive protein (CRP), cystatin C, serum amyloid-A (SAA), tumor necrosis factor-alpha (TNF-α), interleukin-10 (IL-10). Covariates: alcohol, liver fibrosis, comorbidities, CD4 count, antiretroviral therapy, substance use.

**Results:**

Detectable HIV and HCV RNA (OR = 2.49; 95% CI = 1.05–5.89) and detectable HCV RNA alone (2.95; 1.08–8.01) were independently associated with increased odds of having a greater inflammatory burden compared to undetectable viremia. Elevated IL-10 (7.79; 1.90–31.97) and TNF-α (7.70; 1.42–41.83) were independently associated with detectable HIV and HCV RNA. Elevated IL-10 was also associated with detectable HCV RNA alone (5.51; 1.17, 25.84).

**Conclusions:**

Detectable HIV and HCV replication versus undetectable replication was associated with inflammatory burden and certain inflammatory biomarkers independently of alcohol consumption, liver fibrosis and other comorbidities.

## Background

Several reports suggest that human immunodeficiency virus (HIV) infection and hepatitis C (HCV) co-infection with HIV are associated with increased cardiovascular disease (CVD) risk [1-4]. Prior studies also link chronic inflammation, monocyte activation and/or altered coagulation with acute myocardial infarction and death in HIV infected people [5-8]. Whether HIV and HCV mediate their effects on CVD risk through these mechanisms is not known. Assessing whether HIV mono- and HIV/HCV co-infection are associated with increased inflammation is therefore important, though not straightforward. Liver damage caused by alcohol consumption and HCV may alter serum levels of inflammatory biomarkers that are synthesized in the liver (e.g., C reactive protein) and possibly confound the association between viremia and biomarkers of systemic inflammation. Moreover, using a single biomarker, particularly one synthesized in the liver, to represent systemic levels of inflammation may not adequately represent this complex process. Whether a composite measure involving multiple elevated inflammatory biomarkers, including those synthesized in the liver, provides a more complete representation of the state of inflammation in the setting of HIV/HCV infection is not clear.

The objective of this study, therefore, was to examine the association between HIV and HCV viremia and biomarkers of inflammation while accounting for alcohol consumption and liver fibrosis.

## Methods

### Study sample

The HIV-Longitudinal Interrelationships of Viruses and Ethanol (HIV-LIVE) study is a prospective cohort of 400 HIV infected people with current or past alcohol problems. For this analysis, 39 people were excluded because they could not be defined in the HIV/HCV categories: 36 people were missing HIV RNA, 1 person was missing HCV RNA, and 2 people were missing both. Baseline data were collected for the remaining 361 HIV infected participants. As previously reported [9], HIV-LIVE participants were enrolled from four different sources from August 2001 to July 2003: (1) an existing cohort of HIV-infected participants with alcohol problems; (2) Boston Medical Center (BMC)’s Diagnostic Evaluation Unit; (3) Beth Israel Deaconess Medical Center (BIDMC) primary care and specialty clinics; and (4) local health care sites or shelters in the Boston area. Participants were included if they had a positive HIV antibody test (ELISA, confirmed by Western blot), had two or more affirmative responses to the CAGE (Cut down, Annoyed, Guilty, and Eye opener) alcohol screening questionnaire [10] or by physician-investigator diagnosis of alcoholism, spoke English or Spanish, and had at least one contact person likely to know the participant’s whereabouts. Individuals were excluded if the 30-item Folstein Mini-Mental State Examination score [11] was less than 21 or a trained interviewer deemed the patient incapable of comprehending the informed consent or answering interview questions.

### Ethics statement

The Institutional Review Boards of Boston Medical Center, Beth Israel Deaconess Medical Center, and the University of Pittsburgh approved this study.

### Dependent variable

We defined an elevated biomarker as a serum biomarker level >75th percentile for two main reasons. First there are no thresholds associated with clinical events for many of these biomarkers. Second, prior studies of inflammation in HIV infected populations have described associations between biomarker quartiles and parameters of HIV control as well as clinical endpoints such as mortality [8,12].

We examined the following seven biomarkers: interleukin-6 (IL-6), C-reactive protein (CRP), cystatin C, serum amyloid A (SAA), tumor necrosis factor-alpha (TNF-α), monocyte chemotactic protein-1 (MCP-1), and interferon gamma (IFN-γ). The primary outcome, inflammatory burden score, was defined as the presence of zero, one, two, or three or more elevated biomarkers. For example, an inflammatory burden score of 0 corresponds to having none of the seven biomarkers elevated. We defined a score of 3 as having at least three of the seven biomarkers elevated since few people had between four and seven biomarkers elevated. Our secondary outcomes were elevated individual inflammatory biomarkers and included interleukin-10 (IL-10). Importantly, IL-10 was not included in the inflammatory burden score because of its anti-inflammatory properties. For both outcomes, these biomarkers were chosen due to their associations with cardiovascular morbidity and mortality [13-16], HIV [8,17,18], and/or their synthesis in the liver [19,20].

IL-6 was measured by ELISA (Quantiglo Human IL-6 Immunoassay; R&D Systems, Minneapolis, MN) with an assay range of 0.48–1500 pg/mL. The intra-assay and inter-assay coefficients of variation (CVs) ranged from 3.0–5.8% and 6.3–9.6%, respectively. CRP, cystatin C and SAA were measured using a particle enhanced immunonepholometric assay (BNII nephelometer; Dade Behring Inc., Deerfield, IL). The CRP assay range was 0.16–1100 ug/mL. Intra-assay CVs ranged from 2.3–4.4% and inter-assay CVs ranged from 2.1–5.7%. The cystatin C assay range was 0.195–7.330 mg/L. Intra- and inter-assay CVs were <5%. The SAA assay range and minimum detectable level were determined by the lower limit of the reference curve and were dependent on the concentration of the SAA standard used in the assay (N SAA Standard SY). Intra-assay CVs ranged from 4.3–6.2% and inter-assay CVs ranged from 2.8–4.7%. TNF-α, MCP-1, IL-10 and INF-γ were measured using the Human Serum Adipokine Panel B LINCOplex Kit (Linco Research, Inc. St. Charles, MO). The TNF-α and MCP-1 assay ranges were 3.2–50,000 pg/mL. Intra- and inter-assay CVs ranged from 1.4-7.9% and < 21%, respectively. The IL-10 assay range was 3.2–10,000 pg/mL. Intra- and inter-assay CVs ranged from 4.8–9.0% and 3.1–18.4%, respectively. For INF-γ, the assay range was 3.2–10,000 pg/mL. Intra- and inter-assay CVs ranged from 4.8–9.0% and 3.1–18.4%, respectively. All assays were performed at the Laboratory for Clinical Biochemistry Research (University of Vermont, Burlington, VT).

### Independent variable

Detectable viremia was the independent variable categorized into one of four groups: HIV and HCV RNA undetectable (undetectable), HIV RNA but not HCV RNA detectable (HIV mono-detectable), HIV RNA undetectable but HCV RNA detectable (HCV mono-detectable), and HIV and HCV RNA detectable (HIV/HCV detectable). The undetectable group was the referent group for all analyses. HCV RNA was determined from serum collected at the time of enrollment or from participants’ medical records. All participants were HIV antibody positive. HIV RNA testing was performed using a branched-chain DNA assay or polymerase chain reaction (PCR) [21]. The lower threshold of detection was 50–75 copies/mL. All subjects were tested for HCV infection by measuring HCV antibodies and antibody-positive subjects were tested for HCV RNA if these data was unavailable from medical records. HCV RNA was measured either by branched chain DNA or PCR-based assays. The lower level of detection of the assays was 615 IU/mL. HCV antibody-negative subjects were assumed to be HCV RNA negative [22]. This HIV/HCV categorization and the use of viremia (vs. ICD-9 code for example) enable comparisons by HIV and HCV status simultaneously, while minimizing the potential for misclassification bias.

### Covariates

Demographic covariates were age; gender; race (white vs. non-white). Liver fibrosis was assessed as a fibrosis index-4 (FIB-4) score ≥1.45 [23]. We detailed alcohol use data using the 30-day TimeLine Follow Back instrument [24]. Current at-risk alcohol consumption was defined per National Institute on Alcohol Abuse and Alcoholism criteria: drinking >14 standard drinks for men (>seven for women) per week or > four drinks on one occasion for men (>three drinks for women) in the last 30 days [25]. Other covariates were CD4+ T-cell count <200 cells/mm^3^ and self-reported antiretroviral therapy (ART) use at time of assessment, and obesity (body mass index (BMI) ≥30 kg/m^2^). Self-reported comorbid disease was defined as a “yes” response to any of the following questions: “Has a doctor ever told you that you had: CVD (peripheral vascular disease, hardening of the arteries in your neck or legs, atherosclerosis; a stroke, cerebrovascular accident, blood clot or bleeding in the brain, or transient ischemic attack; or a heart attack or myocardial infarction); diabetes or high blood sugar or sugar; hypertension or high blood pressure; high cholesterol; renal disease (poor kidney function or blood tests showing high creatinine); or anemia (low red blood cell count, hemoglobin)?”

For other substance use variables, we defined current smoking as a “yes” response to the question, “Do you currently smoke cigarettes every day or on some days?”; cocaine use as self-reported use of “cocaine, crack or free base”; and injection drug use as a “yes” response to the question, “In your lifetime, have you ever injected drugs?”

### Analysis

Baseline characteristics and biomarker distributions were described and compared by HIV/HCV group using ANOVA, Kruskal Wallis tests, or chi-square tests as appropriate.

The primary analysis (labeled A) used a proportional odds model to estimate the odds of more elevated (>75th percentile) biomarkers. Two models were fit: (1) an unadjusted model with HIV/HCV status only; (2) an adjusted model with HIV/HCV status, age, gender, FIB-4 score, at-risk drinking, CD4 count, ART use, and self-reported comorbidity. The proportional odds model estimates the proportional odds (P_odds_) of having more than *N* concurrently elevated biomarkers versus *N* or fewer. For example, compared to those in the undetectable group, the odds of having more than two versus two or fewer elevated biomarkers is P_odds_ greater for those in the HIV/HCV detectable group. The assumption of proportional odds implies that the coefficients that describe the relationship between an inflammatory burden score of 0 compared to a score of 1 or more are the same as those for an inflammatory burden score of 1 compared to 2 or more. This assumption was assessed by the Score Test. Our secondary analyses used logistic regression to model the odds of having an elevated individual biomarker (labeled B-I) adjusted for the covariates in the models above. Spearman correlation was used to assess potential collinearity in the regression models. No pair of variables within a regression model was highly correlated (r > 0.40). Analyses were conducted using two-sided tests and a significance level of 0.05 and performed using SAS 9.3 (Cary, NC).

## Results

Mean age (range: 40 – 46 years) was different (p < 0.01) across the four HIV/HCV groups and study participants were two-thirds non-white and three quarters male. Evidence of liver fibrosis, diabetes, and CVD was highest among those with HCV whereas at-risk alcohol consumption and immunodeficiency were highest among those with detectable HIV RNA (Table 1).

**Table 1:** Characteristics of 361 HIV-LIVE participants with HIV infection and alcohol problems stratified by detectable HIV and HCV viremia

**Subject characteristics**	**HIV/HCV RNA both undetectable (N = 59)**	**HIV RNA only detectable (N = 122)**	**HCV RNA only detectable (N = 53)**	**HIV/HCV RNA both detectable (N = 127)**	**p-value**
**Age in years, mean (SD)**	43 (7)	40 (8)	46 (7)	43 (7)	<0.01
**Female**	16 (27.1)	26 (21.3)	11 (20.8)	35 (27.6)	0.59
**Race, Non-White**	42 (71.2)	84 (68.9)	34 (64.2)	84 (66.1)	0.84
**FIB-4 ≥ 1.45**	7 (16.3)	28 (28.2)	22 (59.4)	52 (54.7)	<0.01
**FIB-4, Median (Min, Max)**	0.9 (0.5, 3.1)	1.1 (0.2, 7.5)	1.6 (0.7, 14.9)	1.6 (0.4, 17.1)	0.30
**FIB-4, Mean (Standard Deviation)**	1.1 (0.5)	1.3 (1.0)	2.8 (2.9)	2.3 (2.2)	
**Current at-risk alcohol consumption**	15 (25.4)	46 (38.0)	9 (17.0)	43 (33.9)	0.03
**Current CD4+ T-lymphocyte count <200 cells/mm**^**3**^	4 (6.8)	27 (22.9)	6 (11.8)	34 (26.8)	<0.01
**Current antiretroviral medication**	52 (88.1)	56 (45.9)	50 (94.3)	65 (51.2)	<0.01
**BMI ≥30 kg/m**^**2**^	13 (22.8)	23 (19.8)	8 (15.1)	25 (21.2)	0.76
**Prevalent cardiovascular disease**	3 (5.1)	2 (1.6)	6 (11.3)	15 (11.9)	<0.01
**Ever had diabetes**	2 (3.4)	6 (4.9)	9 (17.0)	9 (7.1)	0.02
**Ever had hypertension**	16 (27.1)	28 (23.0)	14 (26.4)	33 (26.0)	0.91
**Ever had high cholesterol**	29 (49.2)	32 (26.2)	11 (20.8)	25 (19.7)	<0.01
**Ever had kidney disease**	2 (3.4)	5 (4.1)	5 (9.4)	9 (7.1)	0.40
**Ever anemic**	17 (28.8)	28 (23.0)	12 (22.6)	31 (24.4)	0.84
**Current smoker**	39 (66.1)	93 (76.2)	40 (75.5)	102 (80.3)	0.22
**Ever cocaine use**	23 (39.0)	53 (43.4)	18 (34.0)	65 (51.2)	0.14
**Ever injection drug use**	10 (17.0)	26 (21.5)	46 (86.8)	115 (90.6)	<0.01
**Ever treated hepatitis C**	0 (0)	3 (2.5)	8 (15.1)	6 (4.7)	<0.01

All values are n (% of HIV/HCV group) unless otherwise specified.

*HIV* human immunodeficiency virus, *HCV* hepatitis C virus, *FIB 4*-liver fibrosis index-4, *BMI* body mass index.

The number of people with available FIB-4 measurements was 43 (HIV/HCV both undetectable), 99 (HIV only detectable), 37 (HCV only detectable), 95 (HIV/HCV both detectable).

**Figure 1:**
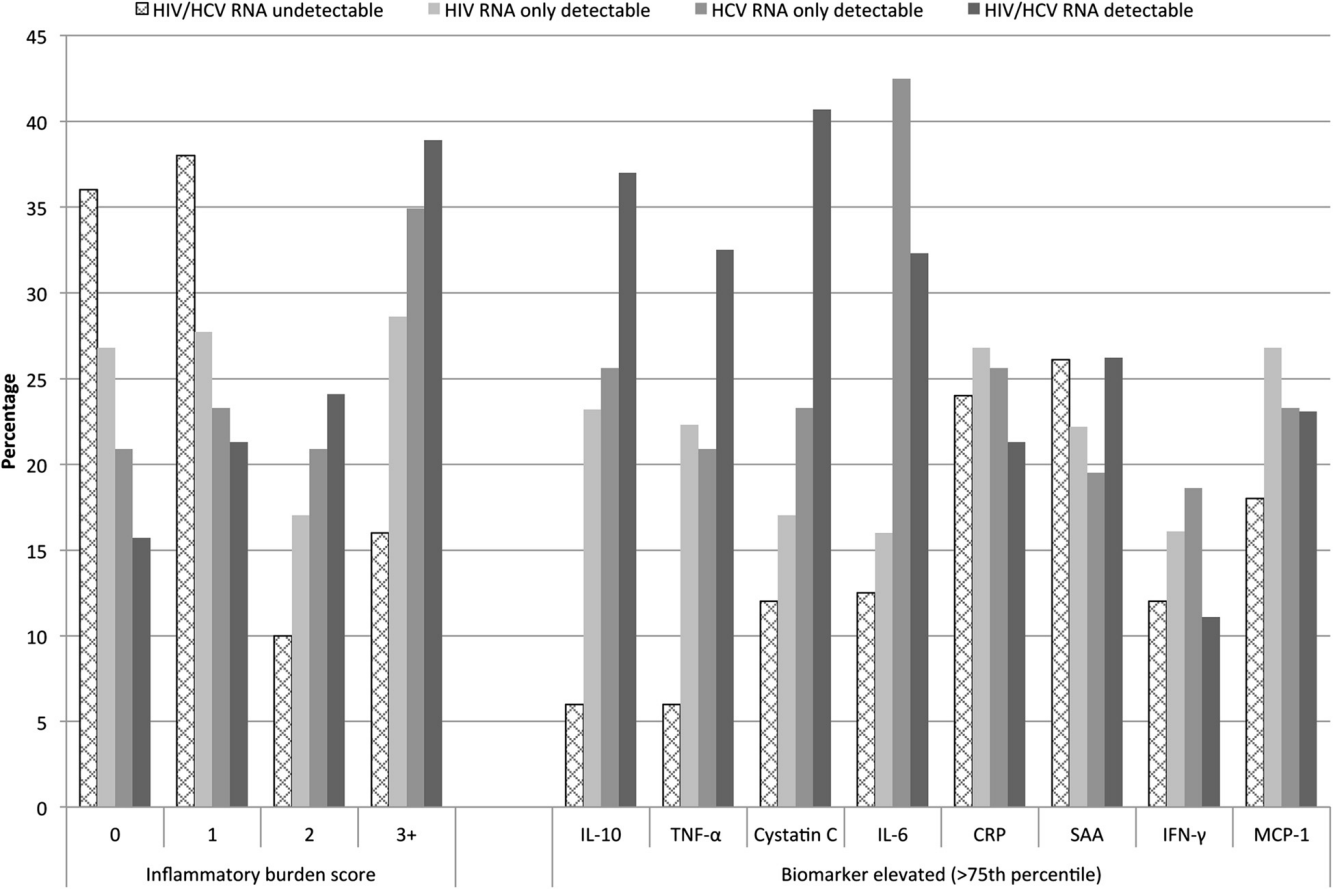
**Inflammatory burden scores (number of elevated biomarkers) and individually elevated biomarkers by HIV/HCV group.** Elevated individual biomarkers were defined as a serum biomarker level >75th percentile. Inflammatory burden score, was defined as the presence of zero, one, two, or three or more elevated biomarkers. For individual biomarkers, the prevalence of elevated IL-10, TNF-α, IL-6 and cystatin C was significantly different across the four HIV/HCV groups (p < 0.05).

Participants in the undetectable group were least likely to have concurrently elevated biomarkers (inflammatory burden score = 2 or 3), while those in the HIV/HCV detectable group were most likely (Figure 1). For individual biomarkers, the prevalence of elevated IL-10, TNF-α, cystatin C, and IL-6 was significantly different across the four HIV/HCV groups (p < 0.05, Figure 1). The highest proportions of elevated IL-10, TNF-α, and cystatin C occurred in the HIV/HCV detectable group (Figure 1). The HCV mono-detectable group had the highest proportion of elevated IL-6 (Figure 1). The prevalence of elevated CRP, SAA, IFN-γ and MCP-1 was similar across the four groups (p > 0.05, Figure 1).

Compared to participants with undetectable HIV and HCV RNA, those in the HIV mono-detectable group (proportional odds ratio (P_OR_) = 1.89 (95% confidence interval (CI) 1.03-3.46), HCV mono-detectable group (P_OR_ = 2.70, 95% CI = 1.29-5.68), and HIV/HCV detectable group (P_OR_ = 3.48, 95% CI = 1.87-6.46) had a significantly higher inflammatory burden (Table 2). This association persisted among participants in the HCV mono detectable and HIV/HCV detectable groups after adjusting for potential confounders (Table 2). FIB4 score ≥1.45 was also significantly associated with an increased burden of inflammatory biomarkers (p < 0.01, Table 2).

**Table 2:** Association of HIV/HCV group with concurrently elevated (>75th percentile) inflammatory biomarkers

**Model A (N = 218)**	**Unadjusted**		**Adjusted**^**a**^	
	**P**_**OR **_**(95% CI)**	**p-value**	**P**_**OR **_**(95% CI)**	**p-value**
**Undetectable**	**1**	**--**	**1**	**--**
HIV mono-detectable	1.89 (1.03, 3.46)	0.04	1.52 (0.68, 3.41)	0.31
HCV mono-detectable	2.70 (1.29, 5.68)	<0.01	2.95 (1.08, 8.01)	0.03
HIV/HCV detectable	3.48 (1.87, 6.46)	<0.01	2.49 (1.05, 5.89)	0.04
FIB-4 ≥ 1.45	--	--	2.56 (1.39, 4.35)	<0.01
High cholesterol	--	--	1.74 (0.97, 3.14)	0.06
Age greater than median (42 yrs)	--	--	0.61 (0.36, 1.04)	0.07
BMI ≥ 30 kg/m^2^	--	--	1.81 (0.93, 3.54)	0.08
Ever smoker	--	--	1.53 (0.85, 2.78)	0.16
CD4 > 200 cells/mm^3^	--	--	0.63 (0.31, 1.26)	0.19
Renal disease	--	--	2.15 (0.67, 6.86)	0.20
Diabetes	--	--	1.90 (0.58, 6.21)	0.29
Current antiretroviral therapy use	--	--	0.74 (0.42, 1.31)	0.30
Prevalent cardiovascular disease	--	--	1.82 (0.56, 5.97)	0.32
Hypertension	--	--	0.78 (0.42, 1.45)	0.44
At-risk alcohol consumption	--	--	0.94 (0.55, 1.62)	0.83
Female	--	--	1.03 (0.55, 1.94)	0.93

^a^Model adjusted for age, gender, FIB-4 score, at-risk drinking, CD4 count, ART use, and self-reported comorbidity.

The ordinal logistic regression model estimates the proportional odds of having more than N concurrently elevated biomarkers versus N or fewer where N = 0, 1, 2, or 3 or more.

*P*_OR_ proportional odds ratio, *HIV *human immunodeficiency virus, *HCV* hepatitis C virus, *FIB* 4-liver fibrosis index-4, *BMI* body mass index, CD4-CD4+ T-lymphocyte count.

Compared to the undetectable group, the HIV/HCV RNA detectable group had higher prevalence of elevated IL-10, TNF-α, IL-6, and cystatin C. After adjustment for potential confounders, this association remained significant for IL-10 (OR = 7.79, 95% CI = 1.90-31.97) and TNF-α (OR = 7.70, 95% CI = 1.42-41.83, Table 3). Elevations in CRP, SAA, IFN-γ, and MCP-1 were not significantly different in any HIV/HCV group compared to the undetectable group in either the unadjusted or adjusted models (Table 3).

**Table 3:** Association of HIV/HCV group with individually elevated (>75th percentile) biomarkers

**Biomarker elevated**	**HIV/HCV group**	**Unadjusted OR (95% CI)**	**Adjusted OR**^**a **^**(95% CI)**
**Model B (N = 218)**	**Undetectable**	1	1
Interleukin-10	HIV mono-detectable	4.74* (1.36, 16.48)	2.95 (0.74, 11.85)
	HCV mono-detectable	5.39* (1.39, 20.84)	5.51* (1.17, 25.84)
	HIV/HCV detectable	9.22* (2.69, 31.55)	7.79* (1.90, 31.97)
**Model C (N = 218)**	**Undetectable**	1	1
Tumor necrosis factor-α	HIV mono-detectable	4.50* (1.29, 15.70)	4.44 (0.86, 22.82)
	HCV mono-detectable	4.15* (1.04, 16.47)	4.45 (0.68, 29.02)
	HIV/HCV detectable	8.50* (2.48, 29.16)	7.70* (1.42, 41.83)
**Model D (N = 196)**	**Undetectable**	1	1
Interleukin-6	HIV mono-detectable	1.33 (0.49, 3.66)	0.57 (0.15, 2.16)
	HCV mono-detectable	5.17* (1.79, 14.94)	2.99 (0.75, 11.98)
	HIV/HCV detectable	3.33* (1.28, 8.70)	1.47 (0.40, 5.35)
**Model E (N = 218)**	**Undetectable**	1	1
Cystatin-C	HIV mono-detectable	1.50 (0.56, 4.01)	0.49 (0.12, 2.02)
	HCV mono-detectable	2.22 (0.73, 6.73)	0.40 (0.07, 2.34)
	HIV/HCV detectable	5.04* (1.98, 12.85)	1.60 (0.39, 6.57)
**Model F (N = 218)**	**Undetectable**	1	1
C-reactive protein	HIV mono-detectable	1.16 (0.54, 2.51)	0.99 (0.35, 2.84)
	HCV mono-detectable	1.09 (0.42, 2.80)	1.41 (0.41, 4.90)
	HIV/HCV detectable	0.86 (0.39, 1.90)	0.69 (0.22, 2.14)
**Model G (N = 210)**	**Undetectable**	1	1
Serum amyloid A	HIV mono-detectable	0.81 (0.36, 1.80)	0.58 (0.19, 1.79)
	HCV mono-detectable	0.69 (0.25, 1.89)	0.38 (0.09, 1.66)
	HIV/HCV detectable	1.01 (0.46, 2.22)	0.72 (0.22, 2.31)
**Model H (N = 218)**	**Undetectable**	1	1
Interferon-γ	HIV mono-detectable	1.40 (0.52, 3.78)	1.65 (0.46, 5.87)
	HCV mono-detectable	1.68 (0.53, 5.28)	2.63 (0.59, 11.76)
	HIV/HCV detectable	0.92 (0.32, 2.60)	1.03 (0.25, 4.30)
**Model I (N = 218)**	**Undetectable**	1	1
Monocyte chemoattractant protein-1	HIV mono-detectable	1.67 (0.72, 3.84)	2.96 (0.80, 10.95)
	HCV mono-detectable	1.38 (0.50, 3.79)	3.71 (0.83, 16.53)
	HIV/HCV detectable	1.37 (0.59, 3.21)	3.48 (0.88, 13.69)

^a^Models adjusted for age, gender, FIB-4 score, at-risk drinking, CD4 count, ART use, and self-reported comorbidity. See Additional file 1: Table S1 for odds ratios for covariates.

*p-value < 0.05.

FIB-4 ≥1.45 was significantly associated with elevated cystatin C (OR = 3.43, 95% CI = 1.45-8.10), IL-6 (OR = 3.22, 95% CI = 1.44-7.20) [26] and MCP-1 (OR = 2.39, 95% CI = 1.10- 5.20). Age above the median (42 years) was associated with lower odds of elevated IL-10, self-reported high cholesterol and diabetes with higher odds of elevated TNF-α, and current ART use with lower odds of elevated TNF-α. Self-reported CVD and smoking were associated with higher odds of elevated cystatin C, and self-reported renal disease with higher odds of elevated SAA and cystatin C. BMI ≥30 kg/m^2^ was associated with higher odds of elevated CRP, and female gender with lower odds of elevated MCP-1 (p < 0.05 for all; Additional file 1: Table S1).

## Discussion

This study suggests that HIV and HCV viremia contribute to elevations in inflammatory burden score, IL-10 and TNF-α, independently of and in addition to the contribution from comorbid conditions. Our results also suggest that a composite measure, comprising multiple inflammatory biomarkers, may suggest an inflammatory state is present even when individual biomarkers do not. The fact that there was no significant association between any HIV/HCV group and CRP or SAA, two biomarkers synthesized in the liver, suggests a need for caution when using these biomarkers to assess inflammation in this population with high potential for liver morbidity.

This study differs from prior work [27-35] in that it attempts to classify people as having more or less inflammation using a concomitantly elevated panel of inflammatory biomarkers rather than emphasizing one individual biomarker of inflammation. Additionally, our results may be more definitive than these prior studies because of our detailed data on alcohol consumption and the inclusion of a validated measure of liver fibrosis.

Findings from our study are consistent with those from prior studies investigating the association between HIV/HCV status and TNF-α [27-30], CRP [31,32], IL-10 [30,33], IFN-γ [30,34,36], IL-6 [28] and cystatin C [35]. However, some differences between our work and these prior studies may reflect different biomarker outcome categorization (quartiles versus detection, secretion, or means), HIV/HCV categorization (viremia versus antibody detection, *in vitro* stimulation with viral proteins) sources of biomarkers (serum versus intrahepatic), referent groups (participants with undetectable HIV and HCV viremia versus HIV or HCV mono-infected participants) and adjustment covariates. The strong associations we reported between detectable viremia and the anti-inflammatory cytokine, IL-10, are consistent with prior research linking persistent viral infection with increased IL-10 production [37]. The lack of data comparing MCP-1, or SAA levels by HIV/HCV status suggests that our findings for these particular cytokines may be novel.

The present study and other work have shown that factors including viral replication, immunocompetence, comorbidity [8,12,18,38], ART [8,18] and ART hepatotoxicity [39], contribute to alterations in some inflammatory biomarkers but not others. If the current study had only investigated CRP, a clinically used inflammatory biomarker [40], we may have concluded that detectable HIV and HCV viremia were not associated with increased inflammation. To more completely describe inflammation, we propose that research into the inflammatory basis of morbidity and mortality should also use composite measures of inflammatory biomarkers. These biomarkers often reflect overlapping biological processes involved in the immune response. Concurrently using multiple biomarkers potentially reduces the variability (intra and inter-person) inherent in measuring and analyzing individual biomarkers. Using single biomarkers to quantify systemic inflammation may be most appropriate in populations with minimal inflammatory comorbid disease. However, a composite measure of inflammation may be more appropriate in HIV-infected populations, where multi-morbidity contributes strongly to chronic systemic inflammation. Future research should compare the contributions of composite versus individual inflammatory biomarker measures to cardiovascular and other end-organ morbidity and mortality prediction.

Strengths of our study include a large, diverse panel of inflammatory biomarkers. Our study sample had well characterized measures of current or past alcohol problems, an important comorbid condition in HIV populations. We had a large proportion of non-white individuals (>50%) enabling generalizability to important minority populations. Limitations that warrant discussion include the lack of HIV uninfected controls, the cross sectional nature of the data presented, self-reported measures of health conditions, and lack of direct cell surface immune activation data for comparison to serum cytokine data. The FIB-4 index is a useful albeit imperfect measure of hepatic fibrosis. This scale was employed as it provides clinically useful prognostic information [23]. We do not expect the index to perform differentially across the four HIV/HCV groups being compared and thus believe that despite its limitations, it is worthwhile to adjust for this covariate to control for potential confounding in assessing the association between HIV/HCV status and inflammatory biomarkers. Sample size limitations may explain the strong but non-significant associations of covariates like high cholesterol, renal disease and smoking with inflammatory burden. This limitation may also explain the stronger association of the HCV mono-detectable group with inflammatory burden versus the HIV/HCV detectable group. As with all observational studies, we cannot exclude the potential for residual confounding.

## Conclusion

In a cohort of HIV infected people with current or past alcohol problems, detectable HIV and HCV RNA compared to undetectable viremia was associated with greater systemic inflammation as measured by an inflammatory burden score and elevations in certain inflammatory biomarkers. This association was independent of at-risk drinking, liver fibrosis and other comorbidities.

## Competing interests

The authors declare that they have no competing interests. Jeffrey Samet, Debbie Cheng, and Matthew Freiberg received NIH/NIAAA grant funding for this work. Russell Tracy’s lab was remunerated for biomarker assays for this study. All other authors had no relevant disclosures.

## Authors’ contributions

JS, MF, RT, JB and KA contributed to the execution/design of the study. EQ and DC provided statistical consulting and performed statistical analyses. KA and MF drafted the manuscript. All authors contributed to critical revision and approved the final version of the manuscript.

## Pre-publication history

The pre-publication history for this paper can be accessed here:

http://www.biomedcentral.com/1471-2334/13/399/prepub

## Supplementary Material

Additional file 1: Table S1 Association of HIV/HCV group and covariates with individually elevated (>75th percentile) biomarkers. Click here for file
