# Supplementary material for: Human immunodeficiency virus, hepatitis C, and inflammatory biomarkers in individuals with alcohol problems: a cross-sectional study
Source: BMC Infect Dis. 2013 Aug 29;13:399. doi: 10.1186/1471-2334-13-399 (PMC3848623; doi:10.1186/1471-2334-13-399)
Supplement: Additional file 1: Table S1 — Association of HIV/HCV group and covariates with individually elevated (>75th percentile) biomarkers. [file 1471-2334-13-399-S1.doc]

Additional file Table 1: Association of HIV/HCV group and covariates with individually elevated (>75th percentile) biomarkers.

|  | **Odds Ratio (95% Confidence Interval)** | | | | | | | |
| --- | --- | --- | --- | --- | --- | --- | --- | --- |
|  | **IL-10** | **TNF-α** | **Cystatin C** | **IL-6** | **CRP** | **SAA** | **IFN-γ** | **MCP-1** |
| **Undetectable** | **1** | **1** | **1** | **1** | **1** | **1** | **1** | **1** |
| HIV mono-detectable | 2.95  (0.74, 11.85) | 4.44  (0.86, 22.82) | 0.49  (0.12, 2.02) | 0.57  (0.15, 2.16) | 0.99  (0.35, 2.84) | 0.58  (0.19, 1.79) | 1.65  (0.46, 5.87) | 2.96  (0.80, 10.95) |
| HCV mono-detectable | 5.51*  (1.17, 25.84) | 4.45  (0.68, 29.02) | 0.40  (0.07, 2.34) | 2.99  (0.75, 11.98) | 1.41  (0.41, 4.90) | 0.38  (0.09, 1.66) | 2.63  (0.59, 11.76) | 3.71  (0.83, 16.53) |
| HIV/HCV detectable | 7.79*  (1.90, 31.97) | 7.70*  (1.42, 41.83) | 1.60  (0.39, 6.57) | 1.47  (0.40, 5.35) | 0.69  (0.22, 2.14) | 0.72  (0.22, 2.31) | 1.03  (0.25, 4.30) | 3.48  (0.88, 13.69) |
| FIB-4 ≥ 1.45 | 1.26  (0.61, 2.62) | 2.07  (0.93, 4.61) | 3.43*  (1.45, 8.10) | 3.22*  (1.44, 7.20) | 1.12  (0.54, 2.32) | 1.13  (0.51, 2.48) | 0.63  (0.25, 1.61) | 2.39*  (1.10, 5.20) |
| High cholesterol | 1.27  (0.58, 2.77) | 4.22*  (1.85, 9.62) | 1.71  (0.71, 4.11) | 0.90  (0.37, 2.17) | 1.20  (0.58, 2.51) | 1.13  (0.50, 2.54) | 1.58  (0.66, 3.80) | 1.81  (0.79, 4.18) |
| Age greater than median (42 years) | 0.42*  (0.20, 0.87) | 0.47  (0.21, 1.02) | 1.05  (0.46, 2.41) | 0.74  (0.33, 1.68) | 0.57  (0.28, 1.14) | 1.82  (0.87, 3.77) | 0.49  (0.20, 1.17) | 0.97  (0.46, 2.05) |
| BMI ≥ 30 kg/m^2^ | 0.57  (0.22, 1.48) | 0.82  (0.32, 2.10) | 1.11  (0.40, 3.08) | 1.37  (0.52, 3.60) | 2.33*  (1.03, 5.24) | 2.05  (0.85, 4.95) | 0.47  (0.15, 1.49) | 1.67  (0.64, 4.38) |
| Ever smoker | 1.02  (0.45, 2.29) | 1.12  (0.47, 2.69) | 3.71*  (1.17, 11.72) | 1.33  (0.52, 3.41) | 1.92  (0.83, 4.45) | 1.57  (0.66, 3.76) | 0.50  (0.20, 1.23) | 0.73  (0.32, 1.65) |
| CD4 > 200 cells/mm^3^ | 1.39  (0.58, 3.34) | 1.72  (0.64, 4.63) | 0.43  (0.16, 1.17) | 0.87  (0.32, 2.38) | 0.65  (0.27, 1.57) | 0.50  (0.20, 1.26) | 1.57  (0.47, 5.26) | 0.53  (0.22, 1.27) |
| Renal disease | 1.24  (0.32, 4.83) | 2.18  (0.54, 8.79) | 9.47*  (2.06, 43.48) | 2.09  (0.45, 9.69) | 1.15  (0.30, 4.35) | 5.17*  (1.23, 21.69) | 2.79  (0.58, 13.38) | 0.14  (0.02, 1.23) |
| Diabetes | 0.91  (0.20, 4.04) | 4.88*  (1.18, 20.21) | 4.91  (0.98, 24.65) | 3.33  (0.69, 16.11) | 3.68  (0.97, 14.02) | 1.26  (0.27, 5.93) | --**  (−−) | 1.08  (0.25, 4.60) |
| Current antiretroviral therapy use | 0.62  (0.29, 1.30) | 0.38*  (0.16, 0.86) | 0.44  (0.18, 1.07) | 0.81  (0.34, 1.92) | 1.04  (0.50, 2.20) | 1.07  (0.49, 2.33) | 1.43  (0.56, 3.65) | 1.30  (0.59, 2.90) |
| Prevalent cardiovascular disease | 0.34  (0.06, 1.84) | 3.64  (0.88, 14.98) | 4.56*  (1.02, 20.43) | 0.32  (0.03, 3.08) | 1.83  (0.44, 7.64) | 1.61  (0.38, 6.89) | 2.73  (0.54, 13.66) | 0.35  (0.06, 2.13) |
| Hypertension | 0.97  (0.42, 2.26) | 0.79  (0.32, 1.92) | 1.09  (0.43, 2.80) | 0.59  (0.22, 1.58) | 0.66  (0.29, 1.51) | 0.51  (0.20, 1.28) | 1.10  (0.41, 2.96) | 0.84  (0.33, 2.08) |
| At-risk alcohol consumption | 0.75  (0.36, 1.55) | 0.80  (0.37, 1.74) | 0.99  (0.43, 2.29) | 1.79  (0.78, 4.08) | 1.06  (0.52, 2.14) | 1.13  (0.53, 2.38) | 1.50  (0.61, 3.65) | 0.63  (0.29, 1.38) |
| Female | 0.79  (0.34, 1.82) | 1.26  (0.53, 3.03) | 1.47  (0.59, 3.66) | 1.21  (0.50, 2.92) | 1.01  (0.45, 2.26) | 1.62  (0.69, 3.80) | 1.36  (0.49, 3.80) | 0.17*  (0.05, 0.60) |

*p-value <0.05;

**There were no elevated IFN-γ among those with diabetes.
